# Friendship Relationship Quality and Cognitive Function: A Cross-Lagged Model of the Role of Depressive Symptoms

**DOI:** 10.1093/geroni/igaf122.2992

**Published:** 2025-12-31

**Authors:** Changmin Peng, Jeffrey Burr

**Affiliations:** University of Massachusetts Boston, Boston, Massachusetts, United States; University of Massachusetts Boston, Boston, Massachusetts, United States

## Abstract

Higher social support and lower social strain from friends have been found to be associated with better cognitive performance. Additionally, adequate cognitive function is considered an essential characteristic for maintaining social interactions with friends in late adulthood. However, research on the phycological mechanisms linking friendship quality and cognitive function is limited. Using data from the 2006-2014 Health and Retirement Study, this study examines the bidirectional relationship between positive and negative friendship quality and cognitive function with depressive symptoms as one potential underlying mechanism among older adults aged 51 and older (N = 6,344). A cross-lagged panel model within the structural equation modeling framework is performed. The results showed that older adults who perceived higher friend support tended to experience fewer depressive symptoms, which in turn, was associated with better cognitive function (indirect effect:b=0.001, p = 0.018). However, the reverse direction was not supported-depressive symptoms was not associated with friend support. Further, older adults who reported more strain with friend reported more depressive symptoms, which in turn, was associated with poorer cognitive function (indirect effect:b=-0.002, p = 0.034). Conversely, older adults with better cognitive function were more likely to report fewer depressive symptoms, which in turn, was associated with less strain with friends (indirect effect:b=-0.003, p = 0.047). These results supported hypotheses that depressive symptoms is one explanation for the bidirectional relationship between friend strain and cognitive function, as well as the unidirectional between friend support and cognitive function. The study has implications for intervention programs aiming at helping older adults stay socially connected and maintain cognitive function.

